# Extract from Dr. C. Von Bœnninghausen’s Preface to His Repertory of the Antipsoric Remedies

**Published:** 1889-03

**Authors:** 


### EXTRACT FROM DR. C. v. BŒNNINGHAUSEN'S PREFACE TO HIS REPERTORY OF THE ANTI-PSORIC REMEDIES.

(Translated from the German by F. H. Lutze, M. D., Cheshire, N. Y.)

* * * As regards the proper size of the dose, I deemed it best at that time to be silent, since Homœopathy has especially of late years learned from experience, to prefer the highest potencies in the smallest doses. Therefore all the better homœopaths use of late only the smallest part of a drop of the highest potencies (*i. e.*, *one*, or at the most *two* of the smallest globules moistened with this potency), and not one of them has had occasion to return to larger doses. There are cases, however, where we are unable to penetrate with such a single dose the disease diathesis and to affect the life-force sufficiently lasting, so as to excite the same to the necessary reaction. To remedy this heretofore existing deficiency without producing by an increase of the size of the dose any unteward effects, and to establish a normal rule, whereby in such cases the smallest dose could be governed so that we might attain our aim with certainty—this remained to be discovered by the latest researches of our worthy and, even in his grand old age, ever busy Hahnemann.* To the noble, gray old man himself I am indebted for the [above] accompanying essay, regarding this nowhere else fully-taught doctrine, which will be none the less acceptable to every homœopath and likewise a great ornament to my humble labor.

*The now deceased Jean Paul Richter used to call him appropriately as well as deservedly, "*A rare double-head of erudition and philosophy*," but he forgot this great man's greatest merit, which, as that of the immortal Linné, indisputably consists in this, that he opened a road, on which the sciences might progress uninterruptedly, daily to enrich the treasures of their experience and to transfer them afterward to the world to come, pure and useful. Just like the botanists, hereafter all physicians in the whole world will understand each other and all will prescribe one and the same remedy for the same symptoms (though not for the same name) of a disease.

